# St. Thomas's and the War

**Published:** 1916-05-06

**Authors:** 


### A THAMES-SIDE MILITARY HOSPITAL.

### St. Thomas's and the War.

Immediately on the outbreak of the war the governors of St. Thomas's Hospital determined to allot all the first-floor wards for the treatment of soldiers. In normal times each of these wards contains twenty-eight beds, with two extra beds in the small wards or on the balcony, but in order that any encroachment upon the amount of accommodation available for the civilian population might be as small as possible it was arranged to bring the number of beds in each up to forty. Anyone who is familiar with these large and airy wards will understand that this increase in the amount of bed accommodation can easily be effected without the merest suspicion of overcrowding. A visit to the wards confirms this. Four of the extra beds have been placed in the centre, and the remainder along each side, and this arrangement still allows of ample space between the beds and more than a sufficiency of air space for each patient even when the wards are fully occupied, which, of course, is not always the case.

### The Problem of the Nursing Staff.

The considerable addition to the number of patients naturally threw a heavy increase of work on the nursing staff, which had already been depleted to some extent, for the war had made claims on a certain number of nurses who had been trained in the hospital and were actually working there at the outbreak of hostilities. The nurses who had gone on active service were replaced, and the staff was supplemented as a result of the return of a number of nurses who had received their training at St. Thomas's and returned from their retirement or from some other duties once more to offer their services to their old hospital. In addition many volunteer ladies who were anxious to help came forward, and have proved a very useful addition to the nursing staff; as they were necessarily untrained and without actual nursing experience, they were distributed among all the wards of the hospital in order that they might go through a course of instruction.

The hospital bore the full expenses entailed by this addition to the nursing staff for the first seven months of the war, when arrangements were made for the extension of the hospital and the addition of a considerable number of extra beds for soldiers. Early in 1915 the War Office had approached the governors with a view to securing the help of the hospital organisation for the treatment and housing of additional military patients. At first it was suggested that an auxiliary hospital should be opened in the neighbourhood, but the opinion of the staff—an opinion in which the governors concurred—was that it would be preferable to erect huts in the quadrangles between the hospital blocks for the accommodation of the additional patients.

### The New Huts.

Accordingly four double huts, one for 66 beds, two for 60 beds each, another for 80 beds, and a single hut of 30 beds, were erected. At the same time quarters were provided for 44 orderlies and an addition was made to the kitchen, while on one other space between the blocks was built a recreation hut for the use of convalescent soldiers. The writer, who recently paid a visit of inspection to these new temporary buildings, obtained the impression that they are as suitable in every practicable way as the wards of the permanent building. Floor and air-space is ample, and the ventilation arrangements are excellent. A criticism that has been profferred is that situated as these huts are between blocks of buildings, they suffer the disadvantage of receiving too much shelter from the sun; at the time of the writer's visit, however, the sun streaming through the windows proved conclusively that this criticism was not altogether sound. The huts are warmed by gas radiators, which in a large measure accounts for the fact that whereas the hospital's gas bill in 1914 was £907, last year it amounted to £1,925.

### Pastimes of Wounded Soldiers.

The recreation room is adequately furnished with comfortable chairs and little tables; a good variety of newspapers and magazines is provided for the soldiers as well as a library of popular novels. There are several small bagatelle and billiard tables in the room, and, of course, card games, chess, draughts, and so on, are played. Perhaps the most interesting feature of this room, however, is the provision made for soldiers who feel disposed to while away the time in some useful occupation. Here, under a skilled instructress, the men may do various kinds of light work such as the making of fancy boxes and picture-frames. The writer was shown quite a presentable flower-pot to which one of the soldiers was giving the finishing touches; this was made of wood with figures in relief covered with the lead sheets taken from a tea-chest, and the maker of this ornament was justly proud of what he told the writer was "his first job." Incidentally, it may be added that it was a soldier from St. Thomas's who took the first prize at the exhibition of wounded soldiers' handicraft. Altogether this recreation room is an excellent institution, and when the warm weather comes and the doors leading to the Embankment promenade can be kept open and the convalescent soldiers can walk in and out, they should be just as happy as the people taking tea on the terrace across the water. No food is served in the recreation room, the patients returning to their own wards at meal-times.

### Five Hundred and Thirty Military Beds.

Now, from September 1914 until the end of February 1915 the governors asked and received nothing from the War Office for what they had done, and it was not until March 1 that arrangements were made for the regulation payment of 4s. per occupied bed—an arrangement that continued until July, when the huts were completed and ready for occupation. The nurses and nursing expenses, however, were paid out of the hospital funds, the result being a very heavily increased demand on the resources of the hospital for the accommodation and feeding of these nurses. This raised a serious question, and while the extension of the hospital was being carried out this and another question—namely, that of the position of the members of the medical staff who were in attendance on the soldiers —had to be made the subject of negotiations with the War Office. The result of these negotiations was that the 530 beds occupied by the military patients were constituted a military hospital known as the 5th London General (City of London) Hospital. The members of the staff were given commissions as from August 16, and the members of the nursing staff working in the military wards became members of the City of London Territorial Forces Nursing Service and were paid independently of the hospital. As the War Office was paying the medical staff and the nurses, the capitation grant was reduced to 3s. per man, the rate per officer remaining as before—namely 5s. 6d. Thirty-three beds, by the way, are allotted to officers, ten in the home, ten in Seymour Ward, and the remainder in small wards. Some idea of what the increase in the nursing staff has meant to the hospital can be imagined when it is stated that whereas in 1914 the average daily number of nurses fed at the hospital was 252, it was 275 in 1915, while up to the present in 1916 it has been 364. These instructive figures include probationers and V.A.D. nurses in the wards. When the hospital was constituted a military hospital the V.A.D. nurses who had been trained in the hospital wards were accepted for service in the military wards as assistant nurses.

### Increase in Expenditure.

It goes without saying that the work St. Thomas's Hospital has done in connection with the war has entailed an enormous increase in expenditure. The rise in the gas bill has already been mentioned, but it will be interesting to give the figures for two other items. The cost of "provisions" in 1914 was £16,120; in 1915 it was £25,906. The cost of coal in 1914 was £4,756, and last year the coal bill was £6,742. In 1914 the average number of patients daily resident in the hospital was 531, last year it was 679.

### Delayed Plans.

In addition to the effects of the war that have just been described the war's influence has been felt in another way. The war has delayed the completion of the plans for the building of the new out-patient department, and this is a matter of serious consequence in view of the great development of new special departments. For instance, the tuberculosis department has now become quite a feature of the hospital, and is of enormous value to South London in general and Lambeth in particular. The Physical Exercise Department, where Swedish remedial exercises and massage are provided for the whole hospital and here instruction is given to a large and growing class of students.

